# The role of anxiety symptoms in school performance in a community sample of children and adolescents

**DOI:** 10.1186/1471-2458-7-347

**Published:** 2007-12-05

**Authors:** Luigi Mazzone, Francesca Ducci, Maria Cristina Scoto, Eleonora Passaniti, Valentina Genitori D'Arrigo, Benedetto Vitiello

**Affiliations:** 1 Division of Child Neurology and Psychiatry- Department of Paediatrics, University of Catania, Catania, Italy; 2 Department of Psychiatry, University of Pisa, Pisa, Italy; 3 Division of Services and Intervention Research, NIMH, Bethesda, MD, USA

## Abstract

**Background:**

Anxiety symptoms are relatively common among children and adolescents and can interfere with functioning. The prevalence of anxiety and the relationship between anxiety and school performance were examined among elementary, middle, and high school students.

**Methods:**

Samples of elementary (N = 131, age 8–10 years), middle (N = 267, age 11–13 years), and high school (N = 80, age 14–16 years) children were recruited from four public schools in a predominantly middle-class community in Catania, Italy. Children completed the Multidimensional Anxiety Scale for Children (MASC). T-scores were computed for the MASC total scores, and considered to be in the anxious range if 65 or above. Current academic grades were obtained from school records.

**Results:**

Of the 478 children, 35 (7.3%) had a MASC T-score in the anxious range. The rate of children in the anxious range was 2.3% in elementary, 7.9% in middle, and 15.9% in high school (χ^2 ^= 7.8, df = 2, p < 0.05), and was 14.1% among students with insufficient grades, 9.4% among those with sufficient grades, and 3.9% among those with good or very good grades (χ^2 ^= 11.68, df = 2, p < 0.01).

**Conclusion:**

In this community sample of children and adolescents attending elementary through high school, the prevalence of abnormally high self-reported levels of anxiety increased in frequency with age and was negatively associated with school performance.

## Background

Anxiety disorders constitute the most common type of psychiatric disorders in childhood. There is, however, considerable variability in the estimated prevalence of these disorders. Based on direct interviews of both children and parents, a three-month rate of 5.7% has been reported among children 9–13 years-old [1] and 2.4% among children 9–16 years-old [2], and a six-month rate of 13.0% of anxiety was found among children 9–17 years-old [3]. In addition, anxiety disorders can be found in the context of other psychiatric disorders, such as mood disorders.

Increasing attention in recent years has been given to "sub-threshold anxiety," namely anxiety which is below the clinical threshold. Indeed, variation of anxiety symptomatology within populations can be better captured using a spectrum approach according to which anxiety is viewed as a continuum of severity encompassing normal as well as pathological behaviour, rather than a disorder based on an arbitrarily derived threshold [4,5].

Anxiety symptoms are extremely common in childhood and adolescence, and can negatively interfere with general well-being, social life, academic performance, and development of social skills [6-12]. Anxiety symptoms are associated with impairment of memory and cognitive functions [13-15], and can contribute to poor school performance and academic failure [16-18], which can, in turn, lead to further psychiatric disturbances [19-21]. Anxiety seems to be an especially important correlate of school failure for girls, whereas disruptive behavioural disorders are more important in males [22]. Anxiety symptoms have been shown to be an important cause of school failure also among males [22,23], and the relative strength of anxiety versus disruptive behavior could be a predictor of boys' and girls' school failure [22]. While poor school performance can result from excessive anxiety, it can also be itself the cause of anxiety, low self-esteem, and other affective symptoms, thus creating a self-maintaining cycle [24-27].

The available data on the link between anxiety and impaired school performance come mainly from clinically referred samples or from surveys of children who had experienced academic failure [21]. Little is known, however, of the possible association between high levels of anxiety symptoms and academic achievement in the community. The main aim of the present study was to evaluate the presence of anxiety symptoms in relation to academic performance in a community sample of children and adolescents attending elementary through high school in a middle-class, urban setting in Sicily, Italy. In order to ensure ecological validity, academic performance was measured with the current actual grades from the school records.

More specifically, the study examined a) the levels of self-reported anxiety symptoms in children and adolescents across different developmental ages (8–16 years) from elementary school through high school and b) the possible association between self-reported anxiety symptoms and school performance. Based on previous reports, it was hypothesized that anxiety would be more prevalent in adolescence than in childhood, and that greater anxiety would be associated with poorer academic achievement.

## Methods

### Sample

Children (age 8–16 years) were recruited from four public schools, including two elementary schools, one middle school, and one high school in a predominantly middle-class urban community in Catania, Sicily (Italy). There were no differences in socio-economic status among the three groups of students. All subjects were Italians. All the contacted schools agreed to participate in the study. The study was approved by the educational board of each school. Overall, 75% of parents of eligible students agreed to participate and all of them gave written informed consent for their children. Only 85% of the high school students returned a completed rating scale. Therefore, the overall final sample consisted of 478 children, age 8–16 years, 222 males (46.3%) and 256 females (53.6%).

The sample included the following three groups according to school level and age:

1) Elementary School (N = 131, age 8–10 years)

2) Middle School (N = 267, age 11–13 years)

3) High School (N = 80, age 14–16 years)

The data were collected between October 2003 and March 2004.

### Assessment

Self-reported anxiety symptoms were assessed using the Multidimensional Anxiety Scale for Children (MASC). This is a 39-item 4-point Likert-style self-report scale with well documented psychometric properties [28]. The MASC is divided into four subfactors: 1) Physical Symptoms, which is further subdivided into two domains exploring respectively tense/restless and somatic/autonomic symptoms; 2) Harm Avoidance, which is further subdivided into two domains exploring anxious coping and perfectionism respectively; 3) Social Anxiety, which is further subdivided into two domains evaluating humiliation/rejection and performance fears, and 4) Separation Anxiety.

The MASC was administered in school to groups of 25 or fewer students. To minimize the influence of individual differences in reading ability, the items were read aloud by an examiner prior to each student completing the questionnaire on her/his own. The examiner was available during the group administration to answer any requests for clarification from the children and to ensure that each child worked independently on her/his questionnaire.

School performance was measured according to the overall grade for each child reported in the official school record at the end of the second quarter of the academic year (ending in March).

Grades were based on a 10-point scale:

1 Very good (≥ 8/10)

2 Good (= 7/10)

3 Sufficient (= 6/10)

4 Insufficient (<6/10)

The teachers' judgment was not taken into account; the global rating for each student was derived from the mean of grades reached in each subject.

### Data Analysis

The MASC total and subfactor raw scores were converted into standard T-scores using the MASC Profile forms for males and for females [25]. T-scores are standard scores with a mean of 50 and a standard deviation of 10. A total MASC T-score equal to or higher than 65 is considered in the anxious range [28]. The χ^2 ^test was applied to analyses of categorical data, such as rates of children with a clinically significant level of anxiety (MASC ≥ 65) and the association between rates of abnormal anxiety and school grades. Continuous variables (MASC total and subfactor scores) were compared across categorical variables using Student's t-test or Analysis of Variance (ANOVA). When the omnibus ANOVA was significant, post-hoc contrasts between pair of groups (Tukey-Kramer HSD) were conducted.

All statistical analyses were conducted on JMP software. A P value of < .05 was used as the threshold for statistical significance. Bonferroni corrections were applied to p's when multiple comparisons were made.

## Results

### Demographics and Anxiety Scores

The demographic characteristics of students and their MASC scores are presented for the elementary, middle and high school participating students in Table 1. Female/male ratios were close to 1 in both elementary (M/F = 65/66) and high schools groups (M/F = 41/39), whereas more females were present in the middle school group (M/F = 116/151). Mean age was 10.49 ± 0.95 in the elementary school (age range: 8–10 years), 12.59 ± 1.07 in middle school (age range: 11–13 years) and 14.07 ± 0.89 in high school (age range: 14–16 years).

**Table 1:** Anxiety symptoms in elementary, middle, and high school students. MASC total and subfactor T- scores (mean ± SE) are compared among the three groups of students.

	Elementary School (E)	Middle School (M)	High School (H)	ANOVA		Post-hoc contrasts
				F	p	
**N**	131	267	80			
**Males**	65	116	41			
**Age (mean ± SD)**	10.49 ± 0.95	12.59 ± 1.07	14.07 ± 0.89			
**MASC Total**	48.20 ± 0.89	48.95 ± 0.62	50.07 ± 1.15	0.8	0.439	
**Physical Symptoms**	50.32 ± 0.87	53.77 ± 0.61	51.44 ± 1.12	5.6	0.003	M>E; M >H;H = E
**Harm Avoidance**	41.03 ± 0.84	41.53 ± 0.59	41.56 ± 1.09	0.12	0.880	
**Social Anxiety**	52.36 ± 0.86	52.73 ± 0.60	53.83 ± 1.15	0.56	0.571	
**Separation/Panic**	45.90 ± 0.87	48.59 ± 0.61	50.05 ± 1.13	4.9	0.008	M>E; M = H; H>E

MASC total scores did not differ significantly among elementary, middle, and high school students (Table 1). Significant differences among these three groups were found only in two MASC subfactors: physical symptoms (ANOVA, p = 0.018) and separation/panic (p = 0.048). Direct contrasts between groups revealed that physical symptoms scores were higher among middle school students than the other two groups. The separation/panic score was lowest among elementary school students, intermediate among middle school students and highest among high school students (Table 1). Gender differences were not found in the MASC total scores (elementary school: p = 0.70; middle school: p = 0.55; high school: p = 0.10) in any groups of students as expected since the T-scores were age- and gender-adjusted.

### Self-reported anxiety symptoms scores in the pathological range

Using the standard MASC cut-off of ≥ 65 in the whole sample, 35 subjects (7.3%) had a pathological level of anxiety and the rate increased from 2% (N = 3 out of 131) in the elementary school group to 7.8% (N = 21 out of 267) in the middle school group and 13.0% (N = 11 of 80) in the high school group (χ^2 ^= 7.8, df = 2, p < 0.05). There were no gender differences in the rates of abnormal scores (7.8% among males and 8.0% among females).

### Relationship between self-reported anxiety symptoms and school performance

There was a statistically significant association between having a MASC total score in the anxious range (i.e., T-score ≥ 65) and poorer academic grades (Table 2). In the whole sample, children with a MASC total score above 65 (N = 35) were more likely to have insufficient school grades (37%) than children with a "non-anxious" score below 65 (18%). This trend was observed within each group of students. More specifically, among elementary students, two out of the three subjects with self-reported anxiety symptoms scores in the anxious range had insufficient grades. In the middle school group, 13 out of the 21 subjects (62%) with self-reported anxiety symptoms scores in the anxious range had insufficient grades.

**Table 2:** Relationship between School Performance and Anxiety Level

School Performance^a^	Anxious range MASC^b ^≥ 65		Normal Range MASC^b ^< 65		All	
	N	(%)	N	(%)	N	(100)
Insufficient	13	(37.1)	79	(17.9)	92	(19.3)
Sufficient	12	(34.3)	115	(26.0)	127	(26.6)
Good – Very Good	10	(28.6)	248	(56.1)	258	(54.1))
All	35	(100)	442	(100)	477	(100)

a. Based on official school grades: a grade of <6 is insufficient, 6 is sufficient, and >6 is good or very good.

b. MASC Total T score.

χ^2 ^= 11.68, df = 2, p < 0.01

Finally, among high school students, all subjects with self-reported anxiety symptoms scores in the anxious range except for one (10/11) had a poor school performance. Across the entire sample, 14.1% (N = 13) of the 92 children with insufficient grades had a MASC total T-score ≥ 65, as compared to 9.4% (N = 12) of the 128 children with sufficient grades and 3.0% (N = 10) of the 258 with good or very good grades (χ^2 ^= 11.68, df = 2, p < 0.01).

Although subjects with an abnormal level of self-reported anxiety symptoms were more likely to have insufficient grades, there was no association between MASC total scores and school performance (ANOVA: elementary school: df = 2, F = 0.38, P = 0.76; middle school: df = 2, F = 0.37, P = 0.76; high school: df = 2, F = 0.68, P = 0.56).

## Discussion

The results of this study, which utilized a community sample of students 8–16 years old, provide evidence of a statistically significant association between high levels of self-reported anxiety symptoms and poor academic performance among students 8–16 years of age. Children with MASC scores considered in the anxious range (i.e., equal or above 65) were more likely to have school grades in the insufficient range (37.1%) than children with scores of 65 or below (17.9%). Our results are in line with those of two previous longitudinal studies [17,29]. Ialongo et al. (1994) found that self-reported anxious symptoms have a significant impact on academic functioning in terms of reading achievement. Grover R et al. (2006) found that highly anxious children were significantly more likely to score lower on measures of academic achievement.

The relationship between self-reported anxiety symptoms and school performance is complex and results from heterogeneous interactions among several factors that include socio-economic status, familiar influences, and individual affective and cognitive profile. School difficulties can be a cause or consequence of affective symptoms and this bi-directional relationship contributes to the maintenance of anxiety and might lead to poor adaptation as well as psychopathologic risk [30-32]. The main implication of these findings is that greater attention should be paid to anxiety symptoms in children and adolescents who are not performing well in school.

Interestingly, in our study, subjects with poor school performance did not display, as a group, a level of self-reported anxiety symptoms (MASC total score) higher than subjects with better academic grades. This result might indicate that anxiety interferes with school functioning only when an abnormal anxiety level is reached, whereas within the "normal" range, being more anxious does not automatically imply worse school functioning and indeed may to a certain extent be motivating and enhancing to academic performance [33]. Of note, the mean MASC total scores were similar across the three groups of students (elementary, middle, and high school), which was expected given the application of age and gender norms to the data. Nevertheless, our data showed that the proportions of subjects with scores in the anxious range (MASC total score ≥ 65) increased with age. Only 2% of elementary school students had an abnormal level of self-reported anxiety, as compared with 7.8% and 13% of middle and high school students respectively. This result is consistent with the onset of common anxiety disorders, such as social phobia and generalized anxiety disorder, which are more likely to occur during adolescence than in childhood and adulthood [34]. Interestingly, our estimates of proportions of participants with anxiety above the clinical threshold (MASC total score ≥ 65) are in the same range as the prevalence of anxiety disorders reported in several epidemiological studies that have used semi-structured/structured interviews rather than self-reported symptoms [3,35]. However, our results should not be compared to those of epidemiological studies because MASC cannot be used to infer formal psychiatric diagnosis.

Anxiety is not the only psychiatric disorder that is associated with impairment of school performance. In the United States, subjects with any psychiatric disorder account for 14.2% of high school dropouts and 4.7% of college dropouts [12]. A wide range of psychiatric disorders appear to underlie this association including depression [36], anxiety disorders [37,38]; obsessive-compulsive disorder [39], attention deficit hyperactivity disorder [40,41], and conduct disorders [38]. Consistent with gender differences in prevalence of internalizing and externalizing disorders, conduct disorders appear to be a major cause of school impairment among males, while anxiety disorders are more important among females [12].

Comparing the two genders for anxiety symptoms, we found that the level of social-phobic and physical symptoms were higher in females than males among students from high and, to lesser extent, middle schools. In contrast, no gender differences were found among primary school students. Our results are in line with previous studies of adolescents that reported higher social anxiety and physical symptoms among females [42,43]. Although this is not a longitudinal study, the comparison among the three different age-groups might, to some extent, inform on the course of anxiety symptoms across the lifespan. Keeping this in mind, our findings suggest that differences in frequencies of social anxiety and physical symptoms between the two genders are likely to appear mainly during adolescence. This result might be explained considering the important hormonal changes that occur in males and females during puberty. Indeed, estrogens have been reported to influence anxiety/social phobic symptoms [44,45] and are very likely to contribute to the development of gender differences in anxiety symptoms.

Results from the current report should be interpreted in the context of several important limitations. The study sample, although representative of the community from which it was drawn, may not necessarily be representative of the general population. The data collected were limited to self-rated anxiety and did not include a comprehensive assessment of other possible symptoms of behavioural or emotional disturbances, such as conduct problems, mood disorders, or substance abuse. The limitation of self-report data is particularly important for the assessment of anxiety among primary school students, because correspondence between informants (parents, children, and teachers) has been found to be consistently low in this age-range. Family socio-economic status was not formally assessed, even though the community where the study was conducted was quite homogeneous and predominantly middle-class. Finally, longitudinal data are necessary to correctly evaluate the development of anxiety symptoms and their consequences during the life-span.

## Conclusion

In conclusion, this study showed that the prevalence of anxiety symptoms increased with age and that high levels of anxiety were negatively associated with school performance in a community sample. Studies to evaluate whether successful resolution of anxiety symptoms result in improved school performance are warranted.

## Competing interests

The author(s) declare that they have no competing interests.

## Authors' contributions

LM designed the study, collected the data, performed statistical analysis and drafted the manuscript.

MCS, EP and VGD, collected the data and assisted in design of the study.

FD and BV commented and participated in the interpretation of data.

All authors read and approved the final manuscript.

## Pre-publication history

The pre-publication history for this paper can be accessed here:


